# Is conventional magnetic resonance imaging superior to radiography in the functional integrity evaluation of anterior cruciate ligament in patients with knee osteoarthritis?

**DOI:** 10.1186/s42836-024-00262-2

**Published:** 2024-06-20

**Authors:** Zhenguo Yu, Hongqing Wang, Xiaoyu Wang, Xin Dong, Jie Dong, Qingchen Liang, Fenglong Sun

**Affiliations:** 1 Department of Orthopedics II, Beijing Rehabilitation Hospital, Capital Medical University, Xixiazhuang, Shijingshan District, Beijing, 100144 China; 2 Department of Dermatology, Peking University Third Hospital, No.49 North Garden Road, Haidian District, Beijing, 100191 China

**Keywords:** Diagnostic validity, Anterior cruciate ligament, Magnetic resonance imaging, Radiography, Knee osteoarthritis

## Abstract

**Background:**

The functional integrity of the anterior cruciate ligament (ACL) influences surgical decision-making in patients with knee osteoarthritis (KOA). This study aimed to compare the diagnostic value of radiography and magnetic resonance imaging (MRI) in determining the functional status of ACL.

**Methods:**

We analyzed 306 knees retrospectively using preoperative hip-to-ankle anteroposterior standing (APS) radiographs, anteroposterior (AP) and lateral knee radiographs, AP valgus stress (VS) force radiographs, and standard orthogonal MRI. Based on the intraoperative visualization, the knees were grouped into ACL functionally-intact and ACL functionally-deficient (ACLD) groups. The diagnostic validity and reliability were calculated based on the radiograph parameters such as hip-knee-ankle angle (HKA), medial proximal tibial angle (MPTA), lateral distal femoral angle (LDFA), posterior tibial slope (PTS), sagittal tibiofemoral subluxation (STFS), coronal tibiofemoral subluxation (CTFS), joint line convergence angle (JLCA), the maximum wear point of the proximal tibia plateau (MWPPT%), and MRI parameters including ACL grades and MWPPT%.

**Results:**

HKA, MPTA, PTS, STFS, JLCA, and CTFS on APS and AP radiographs, and MWPPT% on radiographs and MRI showed a significant diagnostic value (*P* < 0.05). There were no statistically significant differences in the single parameters from radiographs and MRI. After constructing the logistic regression models, MRI showed higher sensitivity, specificity, and accuracy, reaching 96.8%, 79.9%, and 83.3%, respectively (*P* < 0.001).

**Conclusions:**

In patients with KOA, the diagnostic value of single radiographic or MRI parameter in assessing the functional integrity of the ACL are equivalent. However, by constructing predictive models, MRI could significantly improve diagnostic validity compared with radiography.

## Background

Surgical decisions in patients with end-stage knee osteoarthritis (KOA) are directly subject to the functional status of the anterior cruciate ligament (ACL). For patients with anteromedial osteoarthritis (AMOA), a functionally intact ACL is usually considered to be a prerequisite for unicompartmental knee arthroplasty (UKA) [1–3], although no significant difference has been found between ACL-intact and ACL-deficient UKAs in terms of some knee joint functional scores [4]. Surgeons tend to perform total knee arthroplasty (TKA) or UKA in combination with ACL reconstruction in patients with ACL deficiency and antero-posterior instability. This is because tibia anterior migration is increased when ACL is deficient, which leads to accelerated wear and premature failure of the UKA polyethylene component [5, 6]. Therefore, it is crucial to adequately assess the functional status of ACL preoperatively.

Diagnostic accuracy for chronic ACL dysfunction in patients with KOA is poor during physical examination compared with ACL’s acute rupture [1, 7, 8]. Imaging tests have become the most common method for the assessment of ACL integrity, especially with the emergence of magnetic resonance imaging (MRI), and their diagnostic validity has improved significantly. However, in contrast to the numerous studies on the application of radiography and MRI in patients with acute ACL injury, few similar studies have evaluated the functional integrity of ACL in patients with KOA, and no consensus has been reached concerning their diagnostic value.

Kinematically, ACL of knee primarily prevents the tibia from excessive anterior and lateral movements [9]. ACL deficiency causes anterior tibial subluxation, resulting in backward movement of the load-bearing area of the medial tibiofemoral joint, leading to cartilage wear of the posterior tibial plateau [1, 10]. Consequently, the tibiofemoral alignment on sagittal and coronal radiographs is valuable for the assessment of the functional integrity of ACL. Previous investigations showed that, based on the wear range of the medial tibial plateau on lateral radiograph, the sensitivity and specificity of the maximum wear point of the proximal tibia plateau (MWPPT%) in diagnosing ACL integrity were 92.3%–93% and 83.3%–91%, respectively [1, 11]. Altinel et al. [12] demonstrated that the sensitivity and specificity of conventional knee MRI in assessing ACL signal integrity in KOA patients were 73% and 81%, respectively. On the other hand, the subjective judgment of patients with AMOA or posteromedial KOA yielded a sensitivity and a specificity of 36% and 79%, respectively. In addition, an approach of measuring MWPPT% on MRI has not yet been employed in patients with KOA.

The superiority of standard orthogonal MRI scans to radiography in the preoperative ACL diagnosis of patients with KOA remains unclear. Therefore, this study aimed to determine if MRI examination is clinically essential by comparing the sensitivity, specificity, receiver operating characteristic (ROC) curve, and area under the curve (AUC) of various imaging parameters that are possibly associated with ACL dysfunction in its integrity diagnosis, to optimize preoperative examination while ensuring sufficient diagnostic accuracy.

## Methods

We evaluated 306 knee joints of 285 patients who underwent UKA or TKA for KOA between January 2021 and November 2023 at our hospital’s Department of Orthopaedics II. The study was approved by the Ethics Committee for Human Subjects of our hospital (NO. 2023bkky-092). The inclusion criteria were as follows: (1) end-stage medial compartment KOA with Kellgren-Lawrence score ≥ Grade III on radiographs, and (2) knee range of motion ≥ 90° and flexion contracture ≤ 15°. Patients were excluded if they had (1) post-traumatic KOA or history of acute ACL injury, (2) inflammatory comorbidities, such as rheumatoid arthritis, (3) genu valgum or Kellgren-Lawrence score in the lateral compartment ≥ Grade III on anteroposterior (AP) valgus stress radiographs, (4) received previous knee rearrangement surgeries like high tibial osteotomy and distal femoral osteotomy, or (5) incomplete preoperative imaging data or ambiguous surgical records that did not cover ACL functional status. This study’s gold standard was findings from the knee arthroplasty. Patients were grouped into ACL functionally-intact (ACLI) and ACL functionally-deficient (ACLD) groups based on the structural integrity of the ACL under direct visualization and hook examination used to detect ACL tension [13].

Before receiving knee arthroplasty at our center, patients had undergone hip-to-ankle anteroposterior standing (APS) radiography, AP and lateral knee radiography, AP valgus stress (VS) force radiography, and standard orthogonal MRI. When APS radiographs were taken, the patellae were centered within the femoral condyles. The patients bore no weight when the AP and lateral knee radiographs were obtained. The former required the knee joint to be as extended as possible, with the X-rays being perpendicular to the examining table; the latter needed the patient to assume a lateral decubitus position, and the X-ray tube was tilted towards the patient’s head by approximately 7° to ensure an overlap of the bilateral femoral posterior condyles. During the VS radiography, the patients were maintained supine with a 20° knee flexion, and the inspected knee joints were manually subjected to a VS force with a 5°–8° inclination to the head, allowing the X-rays to pass parallel to the joint space. A preoperative knee MRI was performed orthogonally with a 3.0 T field strength. Patients were asked to keep their knee joints extended in the supine position. The axial images were obtained perpendicular to the mechanical axis of the lower limb, with the sagittal images perpendicular to the examination table and the coronal images parallel to the table. The following scanning parameters were set on the MRI facility (GE SIGNA Pioneer, Boston, MA, USA): slice thickness = 3.5 mm, spacing = 0.4 mm, echo spacing = 8.9 ms, pixel size = 0.6 mm × 0.7 mm, including the fat-suppression T2-weighted image.

**Fig. 1:**
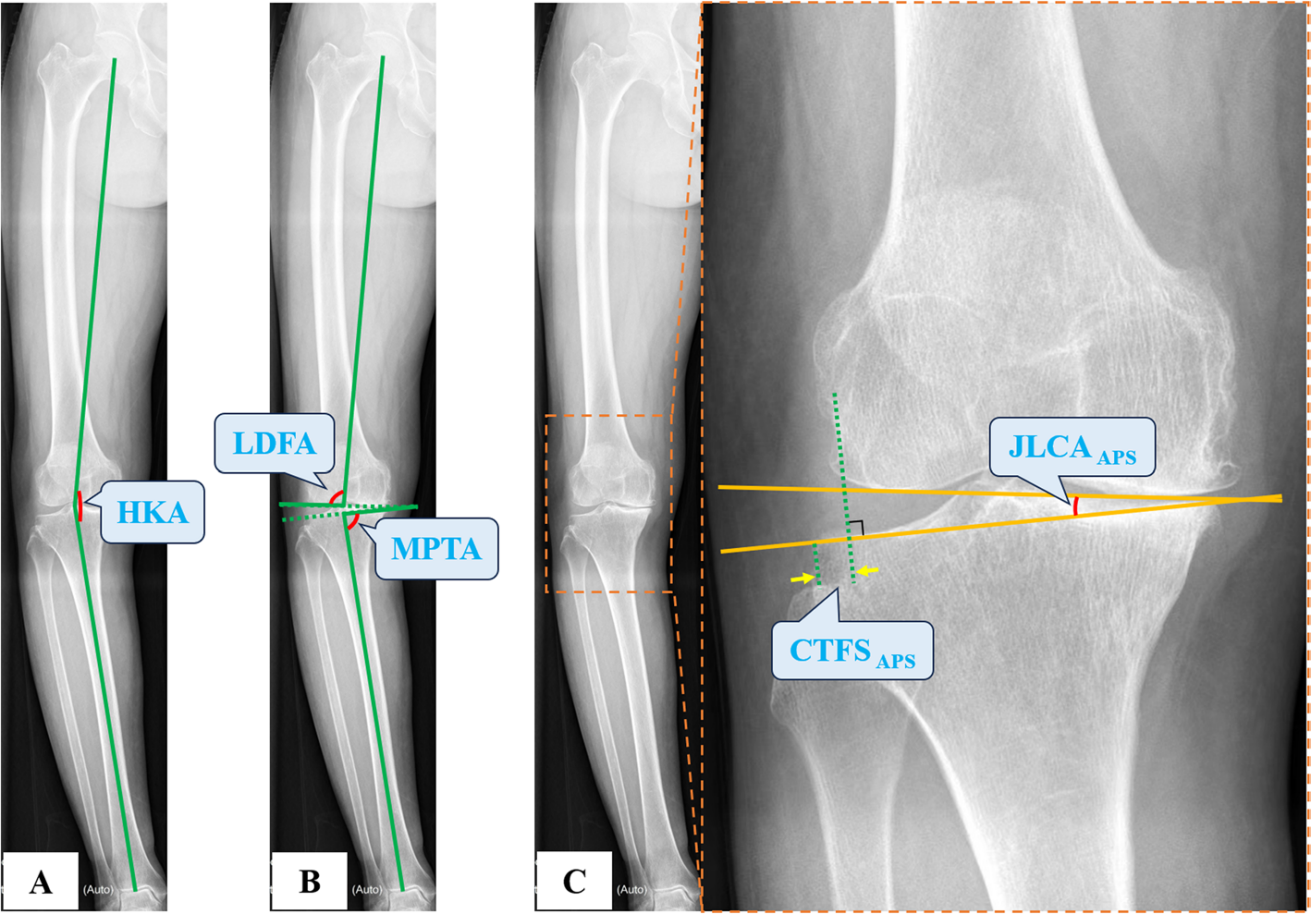
Measurement of parameters on the hip-to-ankle APS radiograph. **A** The midpoints of the femoral head, knee, and ankle joint were connected to form HKA. **B** LDFA was defined as the lateral angle subtended by the distal femoral condylar line and the femoral mechanical axis. MPTA referred to the medial angle between the tibial plateau line and the mechanical axis. **C** On the APS radiograph, CTFS_APS_ was recorded as the distance between the tangent line to the outermost joint edge of the lateral femoral condyle and the utmost margin of the lateral tibial plateau, and JLCA_APS_ referred to the angle between the distal femoral condylar line and the tibial plateau line

**Fig. 2:**
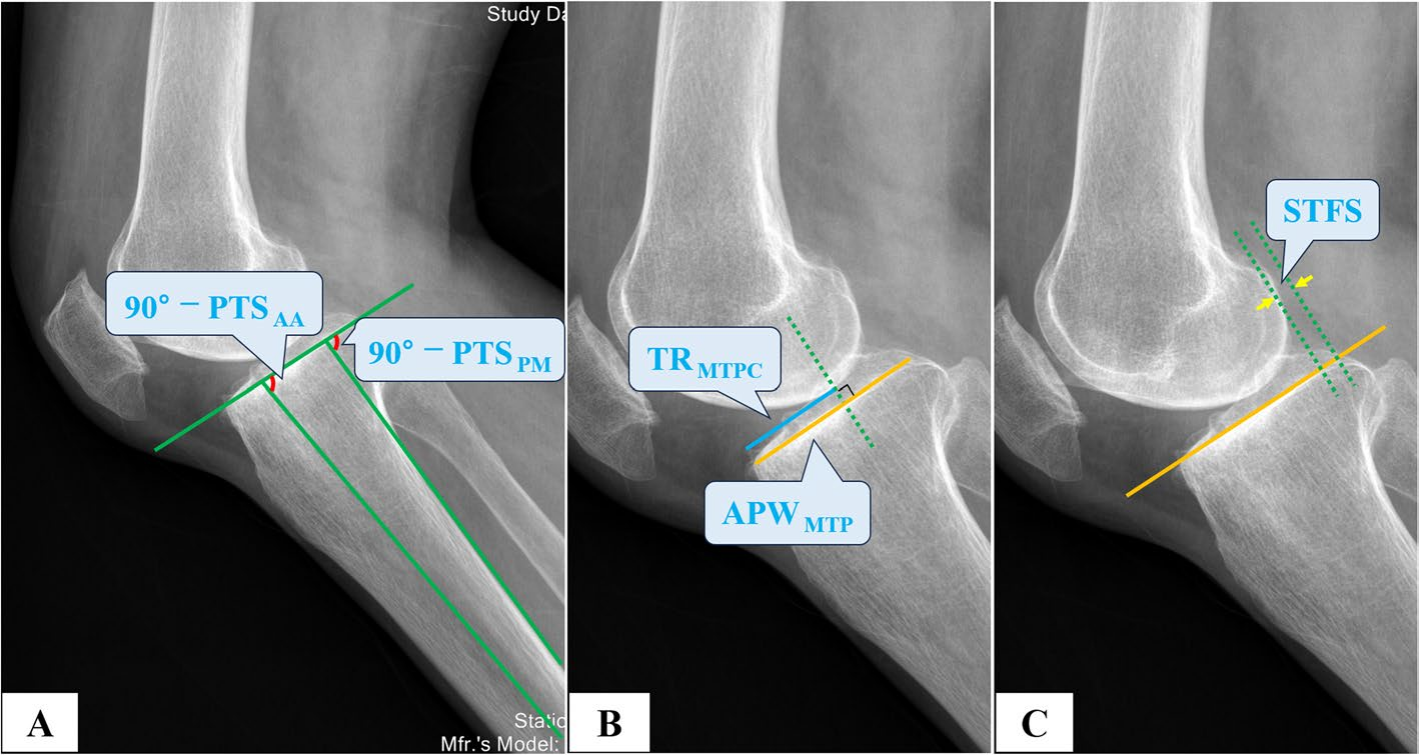
Parameters measured on the lateral knee radiograph. **A** PTS_PM_ referred to the D-value of 90° subtracting the included angle subtended by the posterior margin extension line of the tibial shaft and the medial tibial plateau joint line. PTS_AA_ was defined as the D-value of 90° subtracting the angle between the lateral tibial anatomical axis and the medial tibial plateau joint line. **B** MWPPT%_XR_ was the ratio of the torn range of the medial tibial plateau cartilage (TR_MTPC_) in relation to the AP width of medial tibial plateau (APW_MTP_). **C** Upon drawing a perpendicular line through the most posterior contour of the medial femoral condyle towards the medial tibial plateau joint line, we defined STFS as the distance between the perpendicular line and the most posterior edge of the medial tibial plateau

**Fig. 3:**
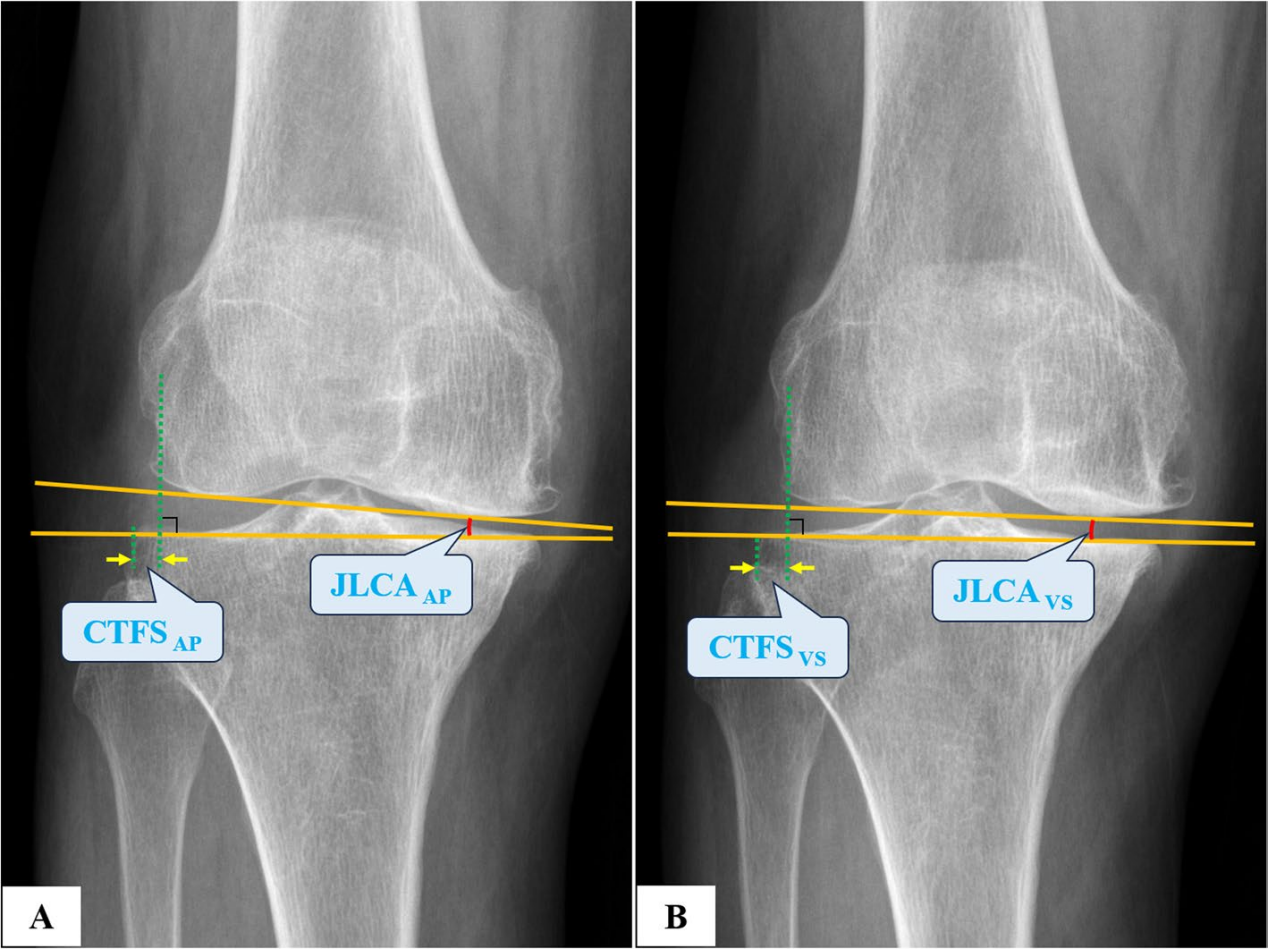
Measurement of parameters on the non-weight-bearing AP knee radiographs. Using the same method shown in Fig. 1C, CTFS and JLCA were measured on regular AP (**A**) and VS radiographs (**B**)

**Fig. 4:**
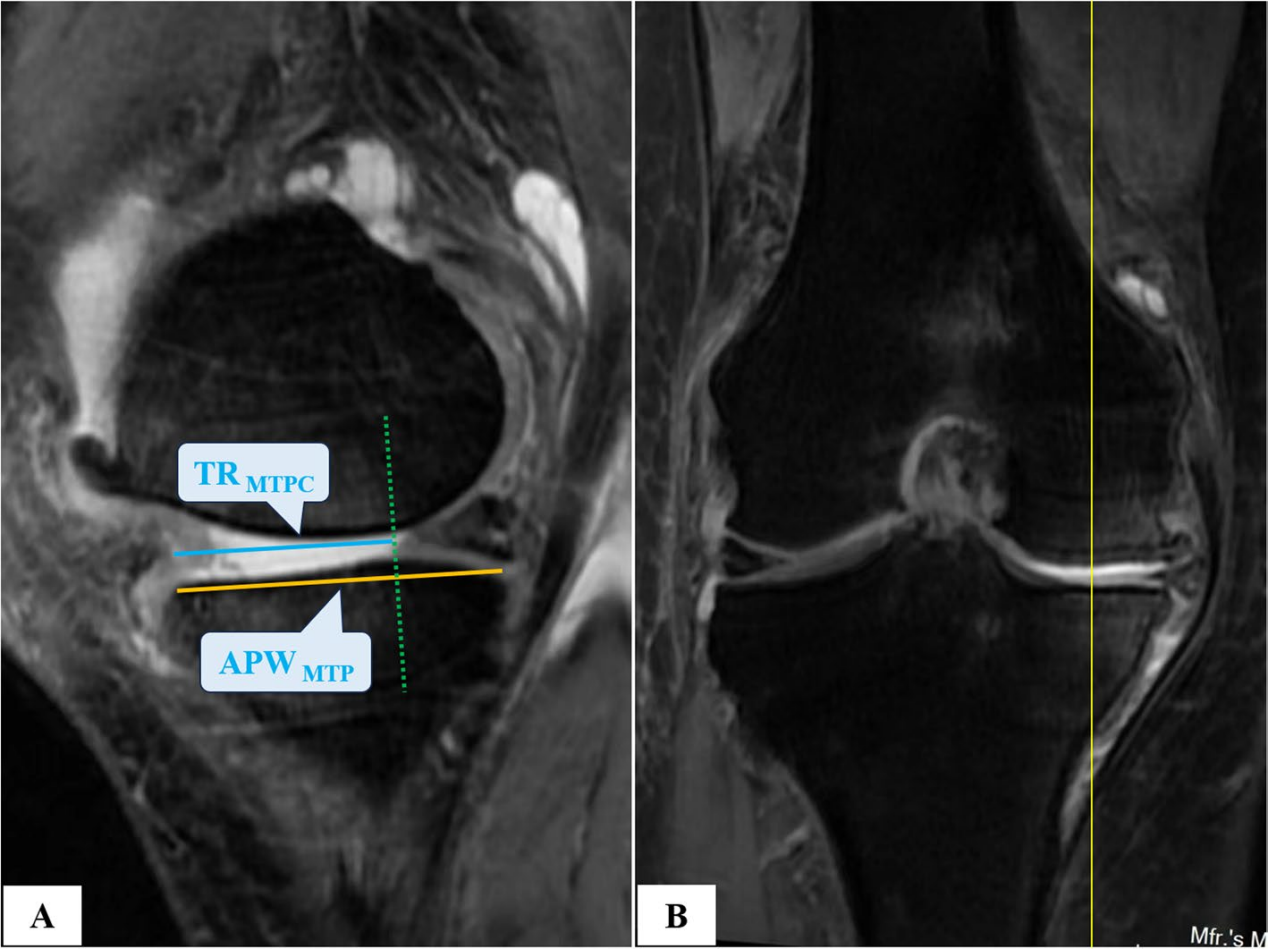
Measurement of parameters on the conventional knee MRI. From the coronal view (**B**), we selected the sagittal section on which the midpoint of the medial tibial plateau was located (**A**) to calculate MWPPT%_MRI_ following the measurement of TR_MTPC_ and APW_MTP_ by the same method for calculating MWPPT%_XR_

Notably, all image parameters were measured and analyzed using an electronic radiology picture archiving and communication system (PACS). The parameters obtained from the hip-to-ankle APS radiographs included the hip-knee-ankle angle (HKA), medial proximal tibial angle (MPTA), and lateral distal femoral angle (LDFA) (Fig. 1A and B). The lateral knee radiographs were used to measure the posterior tibial slope (PTS) and sagittal tibiofemoral subluxation (STFS). PTS was measured using two approaches, with the medial tibial plateau joint line as one edge of the angle. The PTS longitudinal axes were the posterior tibial margin extension line and the line connecting the midpoints of the tibial shaft 5 cm and 15 cm distal to the tibial plateau (lateral anatomical axis). The D-values between the included angles and 90° were represented by PTS_PM_ and PTS_AA_, respectively (Fig. 2A), with a positive value indicating the posterior tilt of the plateau. The ratio of the wear range of the medial tibial plateau (distance from the medial tibiofemoral contact point to the anterior edge of the tibial plateau) to its AP length, as presented by MWPPT%_XR_, was measured and calculated on the lateral radiograph (Fig. 2B). A perpendicular line was drawn through the most posterior contour of the medial femoral condyle towards the medial tibial plateau joint line; the distance between the most posterior edge of the medial tibial plateau and the perpendicular line was referred to as STFS [14] (Fig. 2C), and a positive value was obtained when the medial tibial plateau was located in front of the edge of posterior femoral condyle. In addition, the coronal tibiofemoral subluxation (CTFS) and joint line convergence angle (JLCA) were measured on the hip-to-ankle APS, AP, and VS radiographs and expressed as CTFS_APS_, JLCA_APS_, CTFS_AP_, JLCA_AP_, CTFS_VS_, and JLCA_VS_, respectively (Figs. 1C and 3). The CTFS was defined as the distance between the tangent line to the outermost joint edge of the lateral femoral condyle and the utmost margin of the lateral tibial plateau [15], and CTFS was deemed positive when the former was located more medially to the latter. On sagittal MRI images, grade 1 was indicative of an intact ACL; grade 2 of a partially torn ACL with < 50% ligament substance disruption; grade 3 of a partially torn ACL with a disrupted proportion > 50%; and grade 4 of an absence of ACL [12]. Grades 1–2 ACL were considered functionally intact, whereas those rated grades 3–4 were functionally deficient. On the sagittal MRI image, where the mediolateral midpoint of the medial tibial plateau was located, the percentage of anterior cartilage-worn width in relation to the AP diameter of the medial tibial plateau was calculated and expressed as MWPPT%_MRI_ (Fig. 4). These distance parameters were divided by the patient’s height as the normalized ratio and then multiplied by 160 cm (average height) to generate the calibrated parameters and eliminate the influence of the patient’s height and bone size on the results. Two orthopedic doctors measured or classified these parameters twice, at an interval > 2 weeks.

SPSS (version 22.0) software was used for statistical analysis. A normality test was performed on numerical variables, and *t*-test was utilized to analyze the parameters with a normal distribution, while an X^2^ test was performed on the categorical variables. The validity and reliability of these parameters for assessing ACL functional integrity were calculated separately using diagnostic research method. The validity indices included sensitivity, specificity, Youden index, accuracy, positive likelihood ratio (PLR), negative likelihood ratio (NLR), positive predictive value (PPV), and negative predictive value (NPV). The optimal cut-off value was determined by using the ROC curve method, and the corresponding AUC was calculated for the numerical factors. The reliability index was presented by intra- and inter-observer Kappa values. Sensitivity, specificity, and accuracy were rated as high (≥ 85%), moderate (65%–85%), fair (50%–65%) or low (< 50%) [16]. Diagnostic validity was considered high if the PLR was > 10 or the NLR was < 0.1. The agreement was graded as good (Kappa value ≥ 0.75), moderate (0.4 ≤ Kappa value < 0.75), poor (0 < Kappa value < 0.4), and insignificant (Kappa value ≤ 0) [17]. Statistical significance was set at *P* < 0.05.

## Results

This study examined 306 knees (168 left and 138 right knees) of 285 patients. The ACLI and ACLD groups included 244 and 62 cases, respectively. Table 1 summarizes the basic features of the patients.

**Table 1:** Patients’ basic characteristics

Variables	ACLD group (*n* = 62)	ACLI group (*n* = 244)	*P*-value
Sex (Male/Female)	13/49	55/189	0.790
Age^a^ (years)	67.2 ± 8.7	67.7 ± 6.8	0.625
Weight^a^ (kg)	73.5 ± 13.6	70.7 ± 10.9	0.089
Height^a^ (cm)	160.1 ± 9.9	160.8 ± 7.1	0.524
BMI^b^ (kg/m^2^)	28.7 ± 5.3	27.3 ± 3.3	0.070

^a^The variables were expressed as mean ± standard deviation (SD)

^b^*BMI* Body mass index

With regard to the factors that might affect ACL functional integrity, the two groups showed statistical differences in the mean values of HKA, MPTA, JLCA_APS_, JLCA_AP_, STFS, CTFS_APS_, CTFS_AP_, MWPPT%_XR_, MWPPT%_MRI_, and the MRI grades distribution of the ACL signal. However, no significant differences were found in LDFA, PTS_PM_, PTS_AA_, JLCA_VS_, and CTFS_VS_ (Table 2).

**Table 2:** Differences between the groups’ mean and the distribution characteristics of parameters

Parameters^a^	ACLD group	ACLI group	*P*-value
HKA (°)	167.4 ± 5.0	170.6 ± 3.7	< 0.001
MPTA (°)	84.0 ± 3.6	85.5 ± 2.4	< 0.001
LDFA (°)	89.2 ± 2.8	89.0 ± 2.2	0.538
PTS_PM_ (°)	7.4 ± 3.7	5.7 ± 7.1	0.079
PTS_AA_ (°)	9.9 ± 3.7	8.3 ± 6.6	0.056
JLCA_APS_ (°)	7.1 ± 2.7	5.6 ± 2.4	< 0.001
JLCA_AP_ (°)	4.3 ± 2.3	3.5 ± 1.8	0.004
JLCA_VS_ (°)	1.1 ± 3.0	0.9 ± 2.2	0.605
STFS (mm)	0.2 ± 3.0	-1.0 ± 2.6	0.003
CTFS_APS_ (mm)	8.5 ± 3.0	7.5 ± 2.1	0.001
CTFS_AP_ (mm)	7.3 ± 2.5	6.5 ± 2.0	0.004
CTFS_VS_ (mm)	7.2 ± 3.0	6.6 ± 2.2	0.063
MWPPT%_XR_ (%)	65.6 ± 4.4	59.6 ± 4.9	< 0.001
MWPPT%_MRI_ (%)	81.7 ± 9.9	68.4 ± 8.4	< 0.001
MRI grades of ACL (grade 3–4 / grade 1–2)	60/2	55/189	< 0.001

^a^All the diagnostic parameters were presented as mean ± SD except for the MRI grades of ACL

Table 3 shows the validity of various factors in ACL integrity assessment. The results indicated that HKA, MPTA, PTS_PM_, PTS_AA_, JLCA_APS_, JLCA_AP_, STFS, CTFS_APS_, CTFS_AP_, MWPPT%_XR_, MWPPT%_MRI_, and MRI grades of the ACL were of diagnostic value. However, LDFA, JLCA_VS_, and CTFS_VS_ were of less or limited value in evaluating the ACL functional status.

**Table 3:** Validity of parameters in the diagnosis of ACL functional integrity

Parameters	Cut-off value	Sensitivity (%)	Specificity (%)	Youden index (%)	Accuracy (%)	PLR	NLR	PPV (%)	NPV (%)	AUC [95% confidence interval (CI)]	*P*-value
HKA (°)	169.05	66.1	68.9	35.0	68.3	2.125	0.492	35.0	88.9	0.702 (0.621–0.783)	< 0.001
MPTA (°)	83.05	41.9	84.8	26.7	76.1	2.757	0.685	40.6	85.1	0.640 (0.553–0.727)	0.001
LDFA (°)	-	-	-	-	-	-	-	-	-	0.520 (0.437–0.602)	0.633
PTS_PM_ (°)	8.80	41.9	87.3	29.2	78.1	3.299	0.666	45.6	85.5	0.667 (0.587–0.746)	< 0.001
PTS_AA_ (°)	10.35	50.0	77.9	27.9	72.2	2.262	0.642	36.5	86.0	0.655 (0.575–0.736)	< 0.001
JLCA_APS_ (°)	6.45	56.5	66.8	23.3	64.7	1.702	0.651	30.2	85.8	0.656 (0.579–0.732)	< 0.001
JLCA_AP_ (°)	3.65	61.3	57.4	18.7	58.2	1.439	0.674	26.8	85.4	0.606 (0.525–0.688)	0.010
JLCA_VS_ (°)	-	-	-	-	-	-	-	-	-	0.533 (0.447–0.620)	0.416
STFS (mm)	1.74	35.5	84.8	20.3	74.8	2.336	0.761	37.3	83.8	0.602 (0.518–0.686)	0.013
CTFS_APS_ (mm)	7.48	74.2	51.6	25.8	56.2	1.533	0.500	28.0	88.7	0.631 (0.548–0.714)	0.043
CTFS_AP_ (mm)	8.31	41.9	84.4	26.3	75.8	2.686	0.688	40.6	85.1	0.619 (0.534–0.704)	0.004
CTFS_VS_ (mm)	-	-	-	-	-	-	-	-	-	0.564 (0.479–0.648)	0.121
MWPPT%_XR_ (%)	63.56	74.2	81.1	55.3	79.7	3.926	0.318	50.0	92.5	0.793 (0.731–0.856)	< 0.001
MWPPT%_MRI_ (%)	75.48	85.5	84.8	70.3	85.0	5.625	0.171	58.9	95.8	0.880 (0.823–0.937)	< 0.001
MRI grades of ACL	-	96.7	77.5	74.2	81.4	4.298	0.043	52.2	99.0	0.871 (0.829–0.914)	< 0.001

**Fig. 5:**
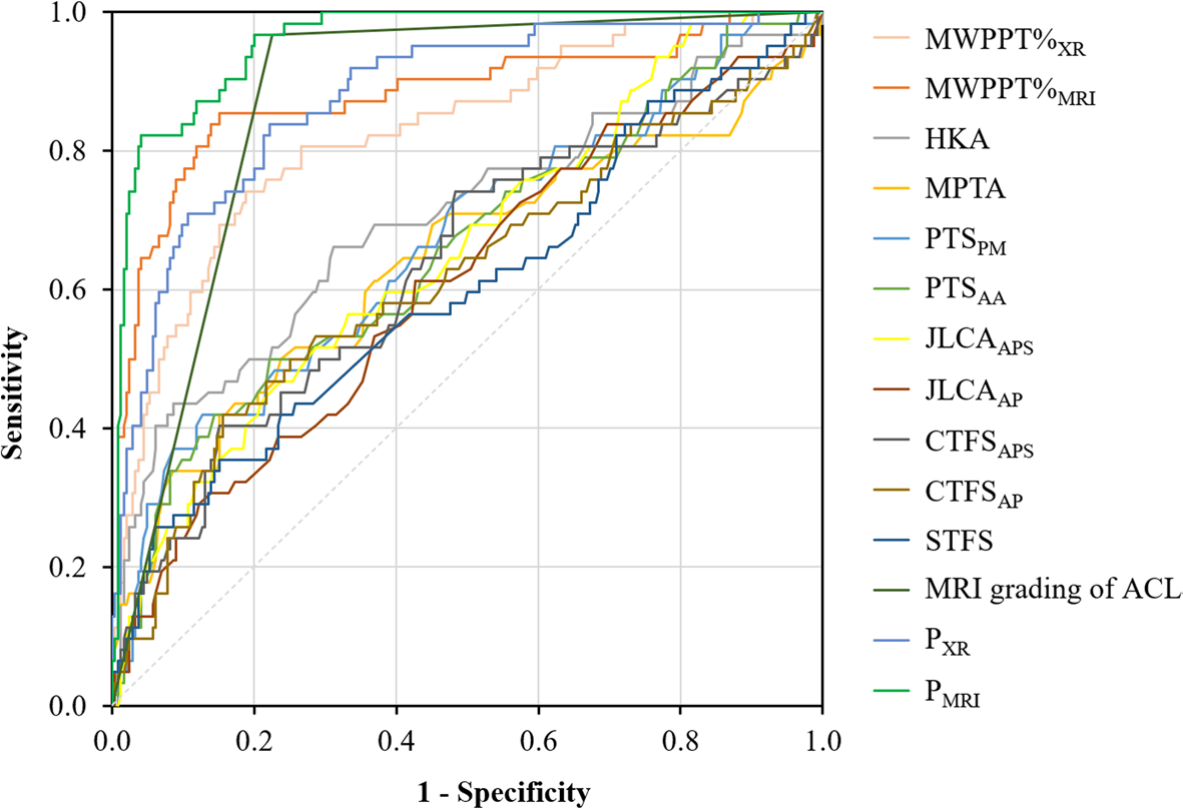
The ROC curve of the parameters for diagnosing ACL functional integrity. Sensitivity and specificity of radiography or MRI in assessing ACL integrity were improved after the construction of logistic regression models compared to single parameter

Using the parameters with diagnostic value (*P* < 0.05) listed in Table 3, we constructed logistic regression models based on the radiographic and MRI findings. Their validity in diagnosing ACL status was also calculated (Table 4). The risk score of the prediction model from the radiographs was Logit(P)_XR_ = 14.579 - 0.013 × HKA - 0.133 × MPTA - 0.002 × PTS_PM_ + 0.051 × PTS_AA_ + 0.268 × JLCA_APS_ - 0.056 × JLCA_AP_ - 0.237 × STFS - 0.147 × CTFS_APS_ + 0.174 × CTFS_AP_ + 0.393 × MWPPT%_XR_. The predicted probability of ACL dysfunction was calculated as follows: P_XR_ = e^Logit(P)XR^/ (1 + e^Logit(P)XR^). Similarly, the risk score of the prediction model based on MRI was Logit(P)_MRI_ = -11.581 + 0.153 × MWPPT%_MRI_ - 3.894 × MRI grades of the ACL (grades 1–2 was expressed as 0, grades 3–4 as 1). The corresponding predictive probability was calculated as P_MRI_ = e^Logit(P)MRI^/ (1 + e^Logit(P)MRI^). The ROC curve in Fig. 5 shows the relationship between the sensitivity and specificity of these parameters. Table 5 lists the intra- and inter-observer Kappa values for these diagnostic parameters.

**Table 4:** Logistic regression model’s validity based on radiographic and MRI results in diagnosing ACL functional integrity

Parameters	Cut-off value	Sensitivity (%)	Specificity (%)	Youden index (%)	Accuracy (%)	PLR	NLR	PPV (%)	NPV (%)	AUC (95% CI)	*P*-value
P_XR_	0.1767	83.9	77.9	61.8	79.1	3.796	0.207	49.1	95.0	0.878 (0.831–0.926)	< 0.001
P_MRI_	0.0761	96.8	79.9	76.7	83.3	4.816	0.040	67.4	98.3	0.957 (0.935–0.979)	< 0.001

**Table 5:** Intra- and inter-observer Kappa values of parameters

Parameters	Intra-observer		Inter-observer	
	Kappa (95% CI)	*P* value	Kappa (95% CI)	*P*-value
HKA (°)	0.898 (0.704–1.000)	< 0.001	0.894 (0.692–1.000)	< 0.001
MPTA (°)	0.762 (0.450–1.000)	0.001	0.659 (0.308–1.000)	0.003
PTS_PM_ (°)	0.828 (0.503–1.000)	< 0.001	0.692 (0.308–1.000)	0.001
PTS_AA_ (°)	0.765 (0.465–1.000)	< 0.001	0.780 (0.492–1.000)	< 0.001
JLCA_APS_ (°)	0.798 (0.533–1.000)	< 0.001	0.706 (0.412–1.000)	0.001
JLCA_AP_ (°)	0.560 (0.180–0.940)	0.012	0.681 (0.352–1.000)	0.002
STFS (mm)	0.500 (0.137–0.863)	0.019	0.529 (0.135–0.923)	0.015
CTFS_APS_ (mm)	0.588 (0.235–0.941)	0.007	0.700 (0.388–1.000)	0.002
CTFS_AP_ (mm)	0.688 (0.286–1.000)	0.002	0.571 (0.142–1.000)	0.010
MWPPT%_XR_ (%)	0.604 (0.263–0.945)	0.006	0.596 (0.241–0.951)	0.008
MWPPT%_MRI_ (%)	0.694 (0.346–1.000)	0.002	0.792 (0.518–1.000)	< 0.001
MRI grades of ACL	0.792 (0.518–1.000)	< 0.001	0.783 (0.505–1.000)	< 0.001
P_XR_	0.800 (0.537–1.000)	< 0.001	0.600 (0.249–0.951)	0.007
P_MRI_	0.667 (0.327–0.994)	0.002	0.733 (0.386–1.000)	0.001

## Discussion

The current study systematically investigated the validity and reliability of radiography and MRI for assessing ACL functional status in patients with KOA using a diagnostic research method. Conventional MRI outperformed radiography upon constructing the predictive models in patients with KOA. However, there was no statistically significant difference in the diagnostic accuracy of a single parameter of radiography and MRI. A functionally-deficient ACL is currently considered to be contraindicated for UKA [18]. Therefore, whenever possible, preoperative MRI should be performed to help evaluate the ACL functional integrity and optimize subsequent operative plans.

With the development of precision medicine in orthopedics, scholars have begun to re-examine the UKA recently. Although TKA instruments are usually prepared routinely before knee arthroplasty to manage intraoperative unexpected conditions such as a deficient ACL, the additional preparation would be redundant if the functional intactness of ACL and full-thickness of lateral tibiofemoral cartilage could be ascertained before surgery. This information saves equipment and personnel resources, since, in some hospitals, the UKA and TKA protheses may come from different companies. Moreover, adequate communication between the surgeons and patients is vital before surgery, and a clearer preoperative plan (including the likelihood of TKA) will help increase patients’ trust in the doctor and compliance with his or her advice.

This study strictly followed the requirements of diagnostic studies in terms of design and implementation by using intraoperative ACL classification as the gold standard. Diagnostic suspicion bias was eliminated during data collection by using a blind method (Only ID numbers were used) in retrieving image data in the PACS. Moreover, there was no verification bias, as the imaging examination conducted preoperatively did not affect the patients’ decision to undergo the subsequent operation. Considering that accuracy and predictive value were influenced by morbidity rate, we continuously included patients who met the inclusion criteria into the cohort, thus retaining the original ACLI-to-ACLD ratio. We found that all the intra- and inter-observer Kappa statistics were > 0.5, indicating that each parameter had moderate repeatability and diagnostic reliability [17]. However, the Kappa values of STFS and CTFS were relatively small (0.5–0.6), which might be ascribed to the increased difficulty in identifying the edges of the tibial plateau and femoral condyle caused by excessive osteophytes in patients with KOA.

In knee kinematics, a functionally-intact ACL limits excessive tibial anterior and lateral movement [9]. Theoretically, the tibiofemoral alignment in the sagittal and coronal views (such as the CTFS, STFS, and MWPPT%) can reflect ACL functional status. Springer et al. [19] reported that CTFS in 64% of patients with ACL deficiency was > 6 mm, but Liu et al. [11] believed that it was insignificant. Notably, the lateral instability after cartilage wear in the weight-bearing area at the knee extension might increase the CTFS of patients with AMOA with a functionally-excellent ACL. Nonetheless, the present study still showed a significant diagnostic value of CTFS_APS_ and CTFS_AP_. Scott et al. [1] reported that the MWPPT% obtained on lateral knee radiographs had the highest sensitivity (93%) and specificity (91%) at a cut-off value of 55%. Similarly, using MRI images plus the Lachman test as the gold standard, Liu et al. [11] recommended an MWPPT% cut-off value of 52.4%, which yielded a sensitivity and specificity of 92.3% and 83.3%, respectively. In our study, we took 63.6% and 75.5% as the cut-off values for MWPPT%_XR_ and MWPPT%_MRI_, respectively and moderate-to-excellent diagnostic validity was achieved; however, the corresponding sensitivity and specificity were inferior to results of the aforementioned studies. This might be attributed to the inconsistent knee flexion angles of lateral radiographs and the increased difficulty in measurement caused by excessive osteophytes.

When the ACL function is intact, though the anteromedial cartilage wear of tibial plateau might exist, the remaining posterior cartilage still maintains the medial collateral ligament (MCL) tension at flexion to avoid contracture. Currently, genu varus can be partially corrected at 20° of knee flexion. However, after ACL failure and posterior wear of the plateau, the MCL contracts as it stays relaxed during knee flexion and extension [10]. Therefore, we are led to speculate that there may be differences in JLCA_VS_ and CTFS_VS_ between ACLI and ACLD groups. However, the results indicated the otherwise, which might be associated with the inconsistent VS force applied to the knee. The JLCA_APS_ and JLCA_AP_ have good diagnostic values. In addition to ACL contracture, knee degeneration may be more severe after ACL dysfunction, and is often accompanied by subchondral bone loss.

It remains unknown if ACL deficiency causes more extensive degenerative changes in patients with KOA or if attritional ACL degeneration is a part of end-stage KOA [20]. However, from a biomechanical perspective, ACL dysfunction induces medial meniscus injury, which further progresses into medial compartmental KOA and varus deformity [19]. Cantin et al. [21] reported that the anterior relaxation caused by ACL deficiency was also associated with KOA progression and genu varus. In addition, excessive traction of ACL caused by lateral thrust during standing and walking is considered one of the reasons for chronic ACL tear in the varus knee [13, 22]. Moreover, varus deformity exacerbates instability, thereby inducing osteophyte proliferation in the intercondylar fossa, which is also crucially responsible for ACL deficiency owing to its cutting effect on the ACL. Therefore, genu varus indirectly reflects the functional integrity of ACL. Springer et al. [19] reported that the ACL deficiency in varus KOA was associated with a larger varus angle, with 73% of patients with ACL dysfunction having a varus angle ≥ 10°. Our study showed that when the HKA cut-off value was set at 169° (11° varus angle), the sensitivity, specificity, and accuracy of ACL status diagnosis were > 65%. In addition, MPTA, an important extra-articular contributor to genu varus, had an independent diagnostic value in our study. Furthermore, some dysplasias, such as a smaller PTS, are considered to be risk factors for ACL rupture [23], which was also validated in the current study.

To date, no consensus has been arrived at concerning whether MRI is superior to radiography in assessing ACL functional integrity in patients with KOA. Altinel et al. [12] suggested that detecting an intact ACL on available radiographs may be sufficient without necessity for additional MRI. Notably, we have also reached the same conclusion by analyzing each diagnostic parameter separately. Among these, ACL grading on MRI yielded excellent sensitivity and moderate specificity. This study showed a much higher sensitivity than specificity with direct ACL imaging, although fibroplasia accompanied by chronic rupture of the ACL in KOA may be misdiagnosed as complete ACL on MRI [24]. This might result from abnormal signals due to the absence of the ACL outer membrane and tissue splitting, which are easily mistaken for ACL deficiency, and the poor visualization was reported in 5–10% of normal ACLs owing to an angular deviation during MRI scan [25]. By constructing predictive models and utilizing the 95%CI of the AUC, we found that MRI had a higher diagnostic value than radiography (*P* < 0.001). The accuracy of this model needs to be further validated in larger samples.

This study had some limitations. First, the knee flexion angles of the lateral radiographs among the subjects ranged from 20°–90°, lacking satisfactory consistency, which might affect the parameters on lateral radiographs, especially STFS. The magnitude of this impact will be elucidated in our subsequent research. Second, VS radiographs were obtained with the assistance of patients’ relatives rather than using a unified stress device, which could not guarantee the consistency of force applied to each subject. Third, the radiographs used in this study did not include lateral radiographs under anterior and posterior stress, which might further improve the validity of radiographs in diagnosing ACL’s functional integrity. Forth, because only a small proportion of the studied patients had ACL deficiency, more samples are required in future studies to reduce the selection bias.

## Conclusions

In patients with KOA, the diagnostic values of single radiography and MRI parameters in assessing the functional integrity of the ACL are equivalent. However, by constructing predictive models, MRI could significantly improve diagnostic validity compared with radiography. Therefore, if the condition permits, preoperative MRI should be performed to evaluate ACL status more precisely and optimize the decision-making about surgery.

## Data Availability

The datasets generated and analyzed during the current study are not publicly available, but are available from the corresponding author on reasonable request.

## Abbreviations

KOA: Knee osteoarthritis
ACL: Anterior cruciate ligament
UKA: Unicompartmental knee arthroplasty
AMOA: Anteromedial osteoarthritis
TKA: Total knee arthroplasty
MRI: Magnetic resonance imaging
MWPPT%: Maximum wear point of the proximal tibia plateau
ROC: Receiver operating characteristic curve
AUC: Area under the curve
AP: Anteroposterior
ACLI: ACL functionally-intact
ACLD: ACL functionally-deficient
APS: Anteroposterior standing
VS: Valgus stress
PACS: Picture archiving and communication system
HKA: Hip-knee-ankle angle
MPTA: Medial proximal tibial angle
LDFA: Lateral distal femoral angle
PTS: Posterior tibial slope
STFS: Sagittal tibiofemoral subluxation
CTFS: Coronal tibiofemoral subluxation
JLCA: Joint line convergence angle
PLR: Positive likelihood ratio
NLR: Negative likelihood ratio
PPV: Positive predictive value
NPV: Negative predictive value
CI: Confidence interval
MCL: Medial collateral ligament
